# Typing of *Yersinia pestis* in Challenging Forensic Samples Through Targeted Next-Generation Sequencing of Multilocus Variable Number Tandem Repeat Regions

**DOI:** 10.3390/microorganisms13102320

**Published:** 2025-10-07

**Authors:** Hyeongseok Yun, Seung-Ho Lee, Se Hun Gu, Seung Hyun Lim, Dong Hyun Song

**Affiliations:** 5th Directorate, 3rd R&D Institute, Agency for Defense Development, Yeuseong P.O. Box 35, Daejeon 34186, Republic of Korea; yunh@add.re.kr (H.Y.);

**Keywords:** *Yersinia pestis*, forensic microbiology, targeted next-generation sequencing, multilocus variable number tandem repeat analysis

## Abstract

Microbial forensics involves analyzing biological evidence to evaluate weaponized microorganisms or their toxins. This study aimed to detect and type *Yersinia pestis* from four simulated forensic samples—human plasma diluted in phosphate-buffered saline (#24-2), tomato juice (#24-5), grape juice (#24-8), and a surgical mask (#24-10). Notably, samples #24-10 may have contained live bacteria other than *Y. pestis*. A real-time polymerase chain reaction confirmed the presence of *Y. pestis* in all samples; however, whole-genome sequencing (WGS) coverage of the *Y. pestis* chromosome ranged from 0.46% to 97.1%, largely due to host DNA interference and low abundance. To address these limitations and enable strain-level identification, we designed a hybridization-based target enrichment approach focused on multilocus variable number tandem repeat analysis (MLVA). Next-generation sequencing (NGS) using whole-genome amplification revealed that the accuracy of the 25 MLVA profiles of *Y. pestis* for samples #24-2, #24-5, #24-8, and #24-10 was 4%, 100%, 52%, and 0%, respectively. However, all samples showed 100% accuracy with target-enriched NGS, confirming they all belong to the same strain. These findings demonstrate that a targeted enrichment strategy for MLVA loci can overcome common obstacles in microbial forensics, particularly when working with trace or degraded samples where conventional WGS proves challenging.

## 1. Introduction

Microbial forensics has evolved as a specialized field dedicated to collecting and analyzing evidence involving microbes or their toxins used in acts of biological crime [1]. It provides a scientific approach to securing evidence from bioterrorism, biological attacks, biological crimes, the intentional manipulation of biological agents and toxins, and the accidental release of such materials [2]. The advent of next-generation sequencing (NGS) has significantly advanced microbial forensics by drastically reducing the time needed for whole-genome analyses of microbial pathogens [3,4]. Consequently, traditional microbial typing methods—DNA fingerprinting, multilocus variable number tandem repeat analysis (MLVA), or multilocus sequence typing (MLST)—can now be performed in silico, while high-precision approaches based on whole-genome sequencing (WGS) enable the identification of single-nucleotide polymorphisms (SNPs) [5,6].

Forensic samples are often limited in DNA quantity, contaminated with exogenous microbial or host DNA present in the environment, or severely degraded [7]. Hybridization-based target enrichment methods have recently been used to analyze the specific genomic sequences of these samples [8,9]. These approaches have been used to capture and sequence ancient DNA from human remains, particularly for challenging samples in which the target DNA may constitute only 1–5% of the total extracted DNA. Target enrichment can increase target DNA yields to over 70% [10,11]. Furthermore, recent studies have successfully used target enrichment to conduct culture-free genomic analyses of hard-to-culture bacteria, achieving high-quality whole-genome sequences from clinical specimens [12]. These findings indicate that NGS with target enrichment holds significant promise for comprehensive analysis of evidence DNA exposed to diverse environmental factors in microbial forensic investigations.

WGS enables high-resolution typing of bacterial pathogens through analyses such as whole-genome SNP and whole-genome MLST (wgMLST), serving as powerful tools in microbial genomics [13,14,15]. The information identifiable only through WGS typically requires pathogen cultivation or highly purified nucleic acids at high concentrations, but NGS may fail to detect or reconstruct genomes when cultivation is unsuccessful or pathogens are present in low copy numbers within the sample [16]. While target enrichment in NGS offers high-resolution bacterial pathogen analysis, its elevated cost and technical complexity may limit the feasibility of targeting multiple pathogens in a single reaction [17]. MLVA is a technique that measures the variation in the number of tandem repeats (VNTR) across multiple loci to determine the genetic relatedness of bacterial strains, offering a relatively straightforward method that provides sufficient resolution for outbreak investigation and epidemiological studies. Despite examining less than 1% of the genome and thus providing lower resolution compared to WGS-based approaches, MLVA remains valuable for comparing microbial sources or assessing equivalence between distinct samples, especially in challenging cases where obtaining reliable WGS data is impractical [18].

Until now, there have been few instances of directly applying target enrichment technology to MLVA profiling. MLVA generally relies on conventional polymerase chain reaction (PCR)-based methods to amplify and analyze specific genetic loci. However, with the advent of NGS, target enrichment approaches such as hybrid capture have been developed, enabling more in-depth analysis of diverse genomic regions. By selectively amplifying or capturing specific genetic targets, these technologies can enhance both the sensitivity and efficiency of the analysis. Consequently, incorporating target enrichment methods into MLVA profiling holds the potential to further improve analytical efficiency and accuracy.

Here, we designed and synthesized target capture probes for the MLVA regions of *Yersinia pestis* to perform typing from trace samples. These samples were provided by the 2024 United Nations Secretary-General’s Mechanism External Quality Assurance Exercise for *Y. pestis* detection and included plasma, tomato juice, grape juice, and a surgical mask containing unknown live bacteria; the other samples were inactivated. Although real-time PCR and NGS revealed the presence of *Y. pestis* in all samples, the chromosome coverage ranged from 0.46% to 97.1% depending on the sample, making strain identification challenging. Using our custom target capture probes, we successfully analyzed the MLVA loci in all samples and confirmed that the *Y. pestis* detected in these four samples belonged to the same strain.

## 2. Materials and Methods

### 2.1. Sample Preparation, Live Sample Handling, and Culture

The test samples were obtained from the Robert Koch Institute and included K2EDTA blood diluted 1:10 in PBS (#24-2), tomato juice (#24-5), grape juice (#24-8), and a punch of a grey surgical mask immersed in 0.8% NaCl, each provided in 0.5 mL volumes. For the commercial juice products, the tomato juice was composed of 99.2% tomato juice from concentrate, 0.5% salt, and lemon juice from concentrate. The grape juice was confirmed to be 100% grape juice. The live sample (#24-10) was provided in a 0.5 mL volume, and 10 μL was inoculated into 10 mL of tryptic soy agar (TSA) broth, followed by incubation with constant shaking at 28 °C for 24 h. All samples were subjected to nucleic acid extraction using the DNeasy Blood & Tissue Kit (QIAGEN) according to the manufacturer’s instructions. For each extraction, 100 μL of the sample was used, and the nucleic acids were eluted in 100 μL of elution buffer. For samples #24-10 and #24-12, 10 μL was spread onto both TSA and cefsulodin–irgasan–novobiocin (CIN) agar plates and incubated at 28 °C for 48 h. The opening and culturing of samples #24-10 were conducted in a biosafety level 3 facility.

### 2.2. Real-Time PCR

For each 20 μL reaction, 2 μL of extracted nucleic acid was combined with 10 μL of 2× TaqMan Gene Expression Master Mix (Applied Biosystems, Foster City, CA, USA), 0.5 μL each of forward and reverse primers (36 μM), 0.5 μL of fluorescent probe (10 μM), and 6.5 μL of double-deionized water, following the TaqPath Master Mix (Thermo Fisher Scientific, Waltham, MA, USA) instructions. Primers and probes were synthesized by Bioneer (Daejeon, Republic of Korea). The probes were labeled with Texas Red (TEX), 6-carboxyfluorescein (FAM), or Cyanine 5 (CY5) and incorporated an internal Bioneer Quencher (i-EBQ) with a phosphate-blocked 3′ end. To detect the pestis chromosome, the *yihN* gene was targeted with forward primer 5′-GCT TTA CCT TCA CCA AAC TG-3′, reverse primer 5′-GAA CCA AAG AAC AAG GA-3′, and probe 5′-[TEX]ATA AGT ACA[i-EBQ] TCA ATC ACA CCG CGA C[Phosphate]-3′. To detect pMT1, the *caf1* gene was targeted using primers 5′-GTT GGT ACG CTT ACT CTT G-3′ and 5′-GTG GTT ATT TCC ATC CTG AG-3′, and probe 5′-[FAM]AAA ACA GGA[i-EBQ] ACC ACT AGC ACA TCT G[Phosphate]-3′. For pPCP1 detection, the *pla* gene was targeted using primers 5′-CTG GTT ACT CCA GGA TGA GA-3′ and 5′-TTC CGG TAT AAG CTC CAT TA-3′, and probe 5′-[CY5]TTG GAC AGC[i-EBQ] TAC AGG TGG TTC ATA T[Phosphate]-3′. All sequences were validated in silico using CLC Genomic Workbench 24. Amplification was performed at 90 °C for 10 min, followed by 40 cycles at 95 °C for 15 s and 60 °C for 1 min on a QuantStudio 6 Flex Real-Time PCR system (Thermo Fisher Scientific).

### 2.3. Whole-Genome Amplification (WGA)

DNA amplification was carried out using the 4BBTM TruePrime^®^ WGA Kit (4basebio, Madrid, Spain) in a reaction volume of 50 μL. The reaction mixture consisted of 2.5 μL of DNA, 2.5 μL of Buffer D, 2.5 μL of Buffer N, 26.8 μL of nuclease-free water, 5 μL of Reaction Buffer, 5 μL of dNTPs, 5 μL of Enzyme 1, and 0.7 μL of Enzyme 2. The thermal cycling conditions were programmed as follows: incubation at 30 °C for 3 h and inactivation at 65 °C for 10 min using a ProFlex thermal cycler (Life Technologies, Carlsbad, CA, USA). The amplified DNA product was then purified using the MinElute PCR Purification Kit (Qiagen, Hilden, Germany).

### 2.4. NGS for Illumina NextSeq

Library preparation was performed using the TruSeq Nano DNA LT Sample Preparation Kit (Illumina, San Diego, CA, USA), following the protocol provided by the manufacturer. DNA samples were fragmented using an M220 Focused-ultrasonicator (Covaris, Woburn, MA, USA). The resulting DNA fragments were size-selected, A-tailed, and ligated to adaptors and indexed primers, followed by enrichment. Sequencing was conducted on the NextSeq benchtop sequencer using the 500/550 Mid Output Kit v2.5 (Illumina).

### 2.5. Construction of the All Living Organisms (ALO) Database and Taxonomic Profiling

To build a metagenome database using all published RefSeq sequences (Archaea, Eukaryota, and Viruses) or bacterial reference genomes available at the National Center for Biotechnology Information (NCBI, https://www.ncbi.nlm.nih.gov/datasets/genome/, accessed on 4 November 2024), we performed domain-based classification and filtering, then downloaded the FASTA files via the FTP server. For each domain, an index for taxonomy profiling was created using CLC Genomics Workbench 24.0. NGS reads were subjected to adapter and quality trimming (>0.05) before taxonomic profiling under default settings. We combined only those families that accounted for ≥1% of reads in each category, designating the rest as Etc. For further NGS read analysis, we retrieved mitochondrial sequences by filtering only the Mitochondrion category from the NCBI organelle database (https://www.ncbi.nlm.nih.gov/datasets/organelle/, accessed on 15 January 2025) and performed taxonomic profiling again.

### 2.6. Target Capture-Based Enrichment for Y. pestis MLVA

Target capture-based enrichment was used for MLVA library preparation of *Y. pestis*. The probe sequences were carefully designed to hybridize specifically to the target bacterial genome. This design involved creating overlapping 120 bp fragments tiled across the MLVA locus, with a 60 bp overlap between consecutive fragments to ensure accurate and efficient target detection. A total of 455 biotinylated probes were developed (Celemics, Seoul, Republic of Korea). Library preparation was carried out using the TruSeq RNA Library Prep for Enrichment kit (Illumina). During the process, DNA was processed, and dual-index adapters (Illumina) were ligated to the fragment ends. Adapter-ligated and amplified libraries were subsequently purified using AMPure XP beads (Beckman Coulter, Brea, CA, USA). The quality and concentration of the libraries were assessed using the TapeStation 4200 system and D1000 ScreenTape (Agilent Technologies, Santa Clara, CA, USA). Final library quantification was performed with the KAPA Library Quantification Kit (KAPA Biosystems, Wilmington, MA, USA) on a QuantStudio 6 Flex Real-Time PCR System (Thermo Fisher Scientific).

### 2.7. Library Preparation and Nanopore Sequencing

The target-enriched library was constructed using the Ligation Sequencing Kit (Oxford Nanopore Technologies, Oxford, UK) per the manufacturer’s protocol. The process, completed within an hour, involved end preparation of the DNA, adapter ligation, and loading onto a FLO-MIN106 (R9.4) flow cell (Oxford Nanopore Technologies, Oxford, UK). Sequencing was performed on the portable MK1C device (Oxford Nanopore Technologies).

### 2.8. MLVA Depth and Analysis

For MLVA, DNA extracted from sample #24-5 was used to prepare a mixture in accordance with the manufacturer’s protocol for nPfu-Special (Enzynomics). PCR conditions included an initial 2 min at 95 °C, followed by 40 cycles at 95 °C for 15 s, 56 °C for 15 s, and 72 °C for 2 min, and a final extension at 72 °C for 2 min, then held at 4 °C. The primers for each locus were chosen based on reference data [19]. PCR products underwent agarose gel electrophoresis, were extracted, and then were subjected to Sanger sequencing using the same primers. Sanger sequencing was conducted on an Applied Biosystems (Life Technologies, Carlsbad, CA, USA) 3500 Genetic Analyzer with the BigDye Terminator v3.1 Cycle Sequencing Kit (Applied Biosystems) and the BigDye XTerminator Purification Kit (Applied Biosystems) per the manufacturer’s instructions. For the 25 MLVA loci, sequences obtained from Sanger sequencing were used as a reference in CLC Genomics Workbench 24.0 to perform read mapping, determine depth, and generate consensus sequences for subsequent MLVA analysis. Short-read reference mapping was performed using the Map Reads to Reference tool with default settings, specifically employing linear gap cost, a match score of 1, a mismatch cost of 2, a length fraction of 0.5, and a similarity fraction of 0.8. Long-read mapping utilized the Map Long Reads to Reference tool with the default Automatic parameter, and no additional specific parameters were determined. Coverage depth was calculated based on the read mapping results using the Quality Control for Targeted Sequencing tool. Consensus sequences for MLVA profiling were then generated using the Extract Consensus Sequence tool based on the read mapping results, setting the low coverage definition threshold at 5 and the low coverage handling method as split into separate sequences to secure the final consensus.

To determine the MLVA profile for each VNTR locus, we first estimated the flanking region size by subtracting the total length of the repeat region (i.e., repeat size multiplied by the number of repeats) from the amplicon size of the *Y. pestis* CO92 MLVA reference. Using this flanking region size, the number of tandem repeats in each sample was calculated by subtracting the flanking region size from the amplicon size inferred from NGS read mapping and then dividing the result by the repeat unit size. The final value was rounded to the nearest whole number and recorded as the repeat copy number for each locus. The MLVA profile of each sample was constructed by compiling the repeat copy numbers across all 25 VNTR loci. The accuracy of the calculated MLVA profiles was assessed through comparisons with validated reference profiles.

## 3. Results

### 3.1. Real-Time PCR and Cultivation Results

Before performing *Y. pestis* real-time PCR on the unknown samples, we validated the performance of the three designed primer-probe sets targeting *yihN*, *caf1*, and *pla*. The limit of detection (LOD) was determined to be 10 copies per reaction (Table S1). Real-time PCR analysis of the four unknown samples (#24-2, #24-5, #24-8, and #24-10) showed that *yihN* had threshold cycle (C_t_) values of 20.12, 24.77, 24.86, and 34.13, respectively, whereas *caf1* displayed C_t_ values of 21.55, 26.39, 26.03, and 34.45, respectively (Table 1). The *pla* gene was not detected in any of the samples. Sample #24-10, which comprised a punch of grey mask in 0.8% NaCl containing live bacteria, was further tested by plating 10 µL onto both TSA and CIN agar plates (Figure S1). Numerous colonies of a single morphology were observed on TSA, but no growth was detected on the *Y. pestis* selective CIN agar plate.

### 3.2. WGA-Based Sequencing and Species Composition

**Figure 1:**
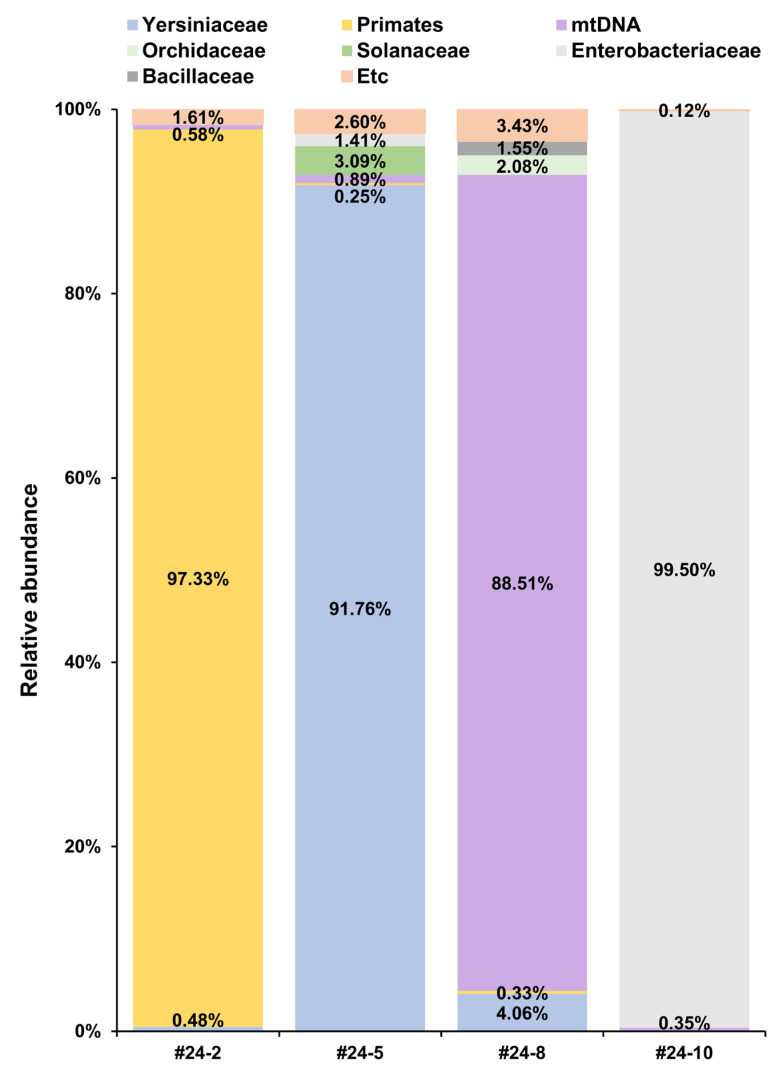
Taxonomic profiling results derived from the All Living Organisms (ALO) metagenomic database and mitochondrial DNA. Percentages below 0.1% are not shown in the graph, and any family contributing less than 1% in each sample is grouped under Etc. Mitochondrial DNA (mtDNA) was identified by re-profiling unmapped reads from the ALO database analysis.

We constructed an ALO metagenomic database using RefSeq sequences from 2169 Archaea, 1885 Eukaryota, and 14,362 Viruses included in the NCBI Genome database and reference genome sequences from 18,640 bacteria included in the NCBI Genome database (Table 2). Since the bacterial RefSeq alone exceeded 1 terabyte in size and contained over 200,000 entries, we used reference genome data instead to avoid memory limitations. After WGA, the four unknown samples (#24-2, #24-5, #24-8, and #24-10) were sequenced with Illumina NextSeq, and the resulting taxonomy profile, showing 90.0%, 84.2%, 9.0%, and 84.6% of the reads from #24-2, #24-5, #24-8, and #24-10 samples, respectively, were mapped to the ALO database (Table S2). Despite this, sample #24-8 exhibited over 90% unmapped reads, prompting de novo assembly of those reads. We identified a 23,810 bp contig to which >70% of #24-8’s unmapped reads aligned, and Basic Local Alignment Search Tool (BLAST, version 2.16.0) analysis confirmed it as mitochondrial DNA (mtDNA). When the NCBI organelle database was added for further taxonomy profiling of #24-8, 69.3% of its total reads were classified as mtDNA. In terms of overall species composition, 91.76% of mapped reads in #24-2 were from *primates*, whereas 0.48% belonged to the *Yersiniaceae* family (Figure 1). For #24-5, 91.76% were from the *Yersiniaceae* family, and 3.09% were from the Solanaceae family, to which tomatoes belong. In #24-8, 88.51% of the mapped reads matched mtDNA, with 61.2% of those related to the fungal genus *Cladosporium*, and 4.06% were from the *Yersiniaceae*. Meanwhile, 99.5% of #24-10’s mapped reads were from the *Enterobacteriaceae* family (primarily *Klebsiella oxytoca*), while no *Yersiniaceae* reads were detected.

### 3.3. Coverage and Depth Analysis with Y. pestis CO92

When reads were mapped to the *Y. pestis* CO92 reference genome, chromosome coverage was 64.3%, 97.1%, 97.0%, and 0.46%, and average depth was 1.1×, 260×, 11×, and 3.8× for samples #24-2, #24-5, #24-8, and #24-10, respectively (Table 3). Plasmid pMT1 was detected in #24-2, #24-5, and #24-8 (coverage: 76.9%, 100%, and 99.9%, respectively; depth: 1.7×, 420×, and 21×, respectively) but not in #24-10. Plasmid pCD1 was found in the same three samples (coverage: 98.0%, 99.9%, and 99.9%, respectively; depth: 5.9×, 1200×, and 56×, respectively), whereas pPCP1 was not detected in any sample.

### 3.4. Target Enrichment and MLVA Profiles

Probes were designed to enrich target regions listed in Table 4, each approximately 120 bp in length and including VNTR sites, and the enriched nucleic acids were then sequenced on a MinION for long-read analysis. When only WGA was used, the fraction of total reads matching the *Y. pestis* CO92 reference was 0.48%, 85.0%, 3.47%, and 0.005% in samples #24-2, #24-5, #24-8, and #24-10, respectively. After target enrichment, however, these proportions rose substantially to 70.9%, 78.4%, 78.7%, and 93.3%, respectively (Table S3). When comparing the number of reads matching the 25 MLVA loci, the proportions during WGA were 0.0004%, 0.062%, 0.002%, and 0% in samples #24-2, #24-5, #24-8, and #24-10, respectively (Table S4). After target enrichment, these proportions increased to 10.15%, 8.72%, 7.79%, and 15.39%, respectively.

**Figure 2:**
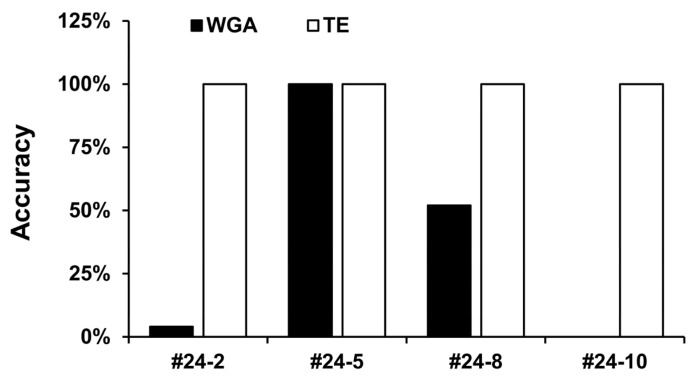
Accuracy of MLVA profiles obtained via whole-genome amplification (WGA) and target enrichment (TE), based on validated reference profiles. MLVA, multilocus variable number tandem repeat analysis.

After target enrichment, the depth of all MLVA loci in samples #24-2, #24-5, and #24-8 was above 80×, and for #24-10, 98.6% of the regions had a depth that was over 80×, while the remaining 1.4% had a depth that was over 40× (Table 5). Contrastingly, when only WGA was performed, 97.4% of the MLVA loci in #24-2 had a depth below 1×, and 100% of regions in #24-10 were below 1×. For #24-5, at least 10× coverage was achieved across all MLVA loci, and 82.4% were above 80× coverage. Sample #24-8 had 34.7% of regions below 1×, 48.7% below 5×, and 16.6% below 20×. In addition, in read depth analysis across the *Y. pestis* chromosome using 10 kb windows, specific enrichment was observed in the MLVA regions under target-enriched conditions (Figure S2). Using the WGA-only approach, the accuracy of MLVA profiling across the 25 loci was 4%, 100%, 52%, and 0% for samples #24-2, #24-5, #24-8, and #24-10, respectively (Figure 2, Table S5). After target enrichment, all 25 MLVA loci were detected in all four samples, resulting in 100% accuracy (Table 6).

## 4. Discussion

In sample #24-5, *Y. pestis* DNA accounted for 85.0% of the total reads, resulting in 97.1% chromosome coverage at a depth of 260× (Table 3). Sample #24-8 contained 3.47% *Y. pestis* DNA, with 97.0% coverage and 11× depth. Despite the difference in read abundance, both samples contained *Y. pestis* genomic DNA at a concentration of 10^6^ genome copies/mL. Notably, both lacked the 102 kb *pgm* locus, which may account for their reduced mapping coverage compared to the *Y. pestis* CO92 reference [20]. Along with the absence of pPCP1, these observations suggest that the isolates are live attenuated *Y. pestis* strains, potentially intended for vaccine use [21].

The C_t_ values of sample #24-2 were lower than those of #24-5 and #24-8, indicating a higher amplification signal. Consistent with this finding, sample #24-2 was confirmed to contain *Y. pestis* genomic DNA at a concentration of 10^7^ genome copies/mL. Interestingly, despite having the higher genome copy number, based on real-time PCR, only 0.48% of total reads matched *Y. pestis* (Table 1 and Table S3). This discrepancy appears to be due to the large amount of human genomic DNA in the sample, a challenge noted in previous studies [22,23,24]. Such issues often necessitate host genome depletion or target enrichment to improve detection sensitivity. Similarly, sample #24-10 exemplifies the diagnostic difficulties posed by trace amounts of non-culturable microbes; the bacterium successfully cultured from this sample was *K. oxytoca* rather than *Y. pestis*.

When we initially received sample #24-10, it was described as a living infectious sample without clear information on whether *Y. pestis* was present. However, we were later informed—after the forensic procedures—that the sample contained live *K. oxytoca* and was spiked with *Y. pestis* genomic DNA at a concentration of 10^7^ copies/mL. The LOD of our *Y. pestis* real-time PCR primers and probes was 10 copies per reaction; the final reaction after TSA enrichment contained approximately 20 genome copies. Although the C_t_ values indicated a weak positive signal, taxonomic profiling did not yield sufficient reads to conclusively identify *Y. pestis*, and reference mapping to the *Y. pestis* CO92 genome showed that only 0.005% of total reads (367 reads) are matched (Table S3). As noted in a previous study, interpreting borderline C_t_ values near the LOD in real-time PCR remains a long-standing challenge, and in our case, it was difficult to make a definitive positive or negative call based on C_t_ values alone [25]. Nonetheless, target-enriched sequencing revealed a clear MLVA profile identical to that of sample #24-5, leading us to conclude that *Y. pestis* of the same strain was indeed present in sample #24-10.

Our study and others have shown that short-read NGS data often produce assembly errors in the VNTR regions used for MLVA [26]. Therefore, we used long-read sequencing on the MinION following MLVA-locus target enrichment. Although indel errors can occur with long reads, they can generally be corrected by increasing the read count [27]. Additionally, to reliably define the true MLVA profile of each sample, we used Sanger sequencing on #24-5—which had the highest *Y. pestis* DNA concentration—as a reference standard. As in earlier reports, we observed a correlation between depth of coverage and MLVA accuracy in the WGA-only short-read data (Table 5, Figure 2). Although the sample quantity was insufficient for a direct comparison of WGA versus target enrichment in short-read NGS, the MLVA-locus depth and resultant MLVA profiles clearly showed that our custom-designed probes worked effectively.

Sample #24-8 was the most intriguing: 91% of its reads were unmatched by our ALO database (Table S3). De novo assembly of the unmapped reads produced a 23,810 bp contig, which accounted for more than 76% of the unmapped reads. BLAST (version 2.16.0) analysis identified this contig as *Cladosporium* spp. mtDNA. After adding the NCBI organelle database and re-running the taxonomic profiling, we observed that 69.3% of the total reads from #24-8 were classified as mtDNA, most of which were *Cladosporium species*. Since *Cladosporium* is a common fungal pathogen in grapes (*Vitis vinifera*), we inferred that #24-8 likely originated from grape juice containing *Cladosporium* mtDNA [28,29,30]. However, no reads were initially assigned to *Cladosporium* in our ALO-based metagenomic analysis.

In general, bacterial target enrichment requires probes covering two to three times the size of the whole genome [31]. Additionally, in a previous study targeting ancient *Y. pestis* DNA, a 120 Mb probe set was used to enable WGS [32]. In contrast, our study demonstrated that strain-level identification of *Y. pestis* is feasible using only 0.055 Mb of probes by targeting MLVA loci specifically. Targeting MLVA loci for enrichment offers a cost-effective and less complex alternative to whole-genome approaches. As the total probe size is minimal, this method has high scalability—allowing, in principle, the simultaneous analysis of hundreds or even thousands of pathogenic species in a single reaction by simply incorporating additional MLVA loci-specific probes. Since MLVA-based enrichment targets <1% of the genome, its resolution is inherently lower than that of WGS, and the possibility of misidentification cannot be completely ruled out [18]. Therefore, for detailed characterization or confirmation, whole-genome target enrichment is still necessary. Rather than replacing such high-resolution methods, the approach we propose serves as a rapid screening tool to provide initial strain-level identification—especially useful in scenarios wherein sample quality or quantity is limited or when quick decision-making is required.

This study presents, to our knowledge, the first strain identification method utilizing target enrichment of MLVA regions for samples containing ultra-low amounts of target DNA. This approach follows the conceptual path of similar strategies in existing literature, where NGS-based detection of biothreat agents has been explored using target amplification of SNP regions [33], and NGS has been successfully performed with target capture of SNP regions on forensically challenging samples [7]. Our ultimate goal is ambitious: to identify hundreds of bacterial pathogens at the strain level in challenging forensic samples, in addition to obtaining whole-genome sequences of specific highly pathogenic viruses via target capture. Given that DNA fragmentation is a likely issue in environmental and forensic samples [7,34], we anticipated that relying on the amplification of numerous amplicons to cover both extensive bacterial targets and complete viral whole-genome sequences would significantly reduce efficiency. Therefore, we chose the target capture approach. While SNP detection undeniably offers higher resolution for detailed strain identification, we intentionally adopted a strategy that sacrifices some of this resolution for compact probe usage to enable efficient, rough strain identification. For example, we used only 455 probes for Yersinia pestis strain identification. We acknowledge, however, that this initial screening approach may necessitate additional sequencing for detailed characterization or confirmation. Consequently, although we considered target amplification of SNP regions for bacteria, as demonstrated in prior literature, the final decision on the optimal workflow must be a comprehensive one, balancing the total number of target bacteria and viruses, the required probe count, and the resulting overall efficiency.

Recent studies have attempted to resolve strain-level variation within specific bacterial species using metagenomic sequencing of clinical and environmental samples [35,36]. Advances in long-read sequencing technologies have also facilitated accurate genome assembly from complex microbiomes, facilitating the separation of closely related strains [37]. However, these approaches typically require deep sequencing coverage to be effective. The MLVA loci-targeted enrichment strategy presented in this study lacks sufficient resolution to distinguish coexisting mixed strains within a single sample. Therefore, while it may serve as a rapid screening tool for strain-level identification, whole-genome target capture and sequencing may still be necessary for precise strain characterization. Future work should also investigate whether this approach can be expanded to simultaneously detect multiple bacterial species using a single probe set.

## Figures and Tables

**Table 1:** Threshold cycle (C_t_) values from real-time PCR for *Yersinia pestis*.

Sample	*yihN*	*caf1*	*pla*
#24-2	20.12	21.55	N.D.
#24-5	24.77	26.39	N.D.
#24-8	24.86	26.03	N.D.
#24-10	34.13	34.45	N.D.

PCR, polymerase chain reaction; N.D., not detected.

**Table 2:** Sources of the All Living Organisms (ALO) metagenomic database.

Groups	Number of Reference Genomes	Source
Archae	2169	NCBI RefSeq
Bacteria	18,640	NCBI Reference genome
Eukaryota	1885	NCBI RefSeq
Viruses	14,362	NCBI RefSeq

NCBI, National Center for Biotechnology Information.

**Table 3:** Coverage and depth of the *Yersinia pestis* CO92 chromosome and plasmids based on WGA-NGS reads.

Sample	Chromosome	pMT1	pCD1	pPCP1
#24-2	64.3% (1.1×)	76.9% (1.7×)	98.0% (5.9×)	N.D.
#24-5	97.1% (260×)	100% (420×)	99.9% (1200×)	N.D.
#24-8	97.0% (11×)	99.9% (21×)	99.9% (56×)	N.D.
#24-10	0.46% (0.1×)	N.D.	N.D.	N.D.

Note: Numbers indicate coverage (%), and read depth is indicated in parentheses (×). WGA-NGS, whole-genome amplification-based next-generation sequencing; N.D., not detected.

**Table 4:** Characteristics of 25 MLVA loci and probe positions in the *Yersinia pestis* CO92 genome.

Locus Name	Repeat Unit Size (bp)	Number of Repeats	Amplicon Size (bp)	Product Locus in Chromosome	Probe Locus in Chromosome
YPO0120ms01	18	8	228	120,578–120,805	120,424–120,959
YPO1290ms04	17	8	230	1,290,058–1,290,287	1,289,906–1,290,439
YPO1935ms05	17	11	291	1,935,743–1,936,033	1,935,630–1,936,146
YPO2769ms06	60	8	606	2,769,255–2,769,860	2,769,157–2,769,957
YPO2916ms07	10	9	184	2,916,119–2,916,302	2,915,951–2,916,470
YPO3057ms09	18	33	682	3,057,920–3,058,601	3,057,768–3,058,753
YPO0559ms15	15	10	237	559,832–560,068	559,700–560,199
YPO1814ms20	15	9	253	1,814,316–1,814,568	1,814,185–1,814,699
YPO1895ms21	18	9	278	1,895,310–1,895,587	1,895,199–1,895,698
YPO4042ms35	15	8	204	4,042,357–4,042,560	4,042,216–4,042,700
YPO4425ms38	16	8	233	4,425,264–4,425,496	4,425,114–4,425,645
YPO0581ms40	17	7	214	581,285–581,498	581,142–581,641
YPO0718ms41	17	7	217	718,740–718,956	718,581–719,114
YPO1018ms44	17	7	233	1,018,592–1,018,824	1,018,459–1,018,958
YPO1108ms45	12	7	161	1,108,932–1,109,092	1,108,817–1,109,268
YPO1335ms46	7	5	112	1,335,216–1,335,327	1,335,027–1,335,526
YPO2058ms51	21	2	207	2,058,613–2,058,819	2,058,456–2,058,976
YPO2612ms54	22	7	281	2,612,645–2,612,925	2,612,525–2,613,046
YPO3060ms56	16	7	220	3,060,490–3,060,709	3,060,350–3,060,849
YPO4280ms62	9	7	124	4,280,809–4,280,932	4,280,606–4,281,132
YPO1118ms69	16	6	179	1,118,954–1,119,132	1,118,786–1,119,301
YPO1580ms70	9	6	146	1,580,056–1,580,201	1,579,866–1,580,392
YPO1925ms71	14	6	171	1,925,682–1,925,852	1,925,511–1,926,024
YPO3236ms73	18	6	225	3,236,766–3,236,990	3,236,601–3,237,154
YPO3245ms74	15	6	195	3,245,551–3,245,745	3,245,399–3,245,898

MLVA, multilocus variable number tandem repeat analysis.

**Table 5:** Distribution of coverage depth (%) for 25 *Yersinia pestis* MLVA loci under whole-genome amplification (WGA) and target enrichment (TE) conditions.

Depth	#24-2		#24-5		#24-8		#24-10	
	WGA	TE	WGA	TE	WGA	TE	WGA	TE
<1×	97.4%	0%	0%	0%	34.7%	0%	100%	0%
5×	2.6%	0%	0%	0%	48.7%	0%	0%	0%
10×	0%	0%	0.6%	0%	16.6%	0%	0%	0%
20×	0%	0%	3.4%	0%	0%	0%	0%	0%
40×	0%	0%	13.6%	0%	0%	0%	0%	1.4%
80×	0%	0%	8.2%	0%	0%	0.1%	0%	1.3%
>80×	0%	100%	74.2%	100%	0%	99.9%	0%	97.3%

MLVA, multilocus variable number tandem repeat analysis.

**Table 6:** Comparison of MLVA profile accuracy across sample matrices using whole-genome amplification (WGA) and target enrichment (TE).

Sample	Matrices	Enrichment	MLVA Profie Accuracy
#24-2	K2EDTA blood diluted 1:10 in PBS	WGA	4%
		TE	100%
#24-5	Tomato juice	WGA	100%
		TE	100%
#24-8	Grape juice	WGA	52%
		TE	100%
#24-10	punch of a grey surgical mask immersed in 0.8% NaCl	WGA	0%
		TE	100%

MLVA, multilocus variable number tandem repeat analysis.

## Data Availability

All raw NGS data used in this study have been deposited in the NCBI BioProject under the accession number PRJNA1226997 (https://www.ncbi.nlm.nih.gov/bioproject/PRJNA1226997/, accessed on 4 October 2025).

## Supplementary Materials

The following supporting information can be downloaded at: https://www.mdpi.com/article/10.3390/microorganisms13102320/s1. Figure S1. Culture results for sample #24-10. A total of 10 µL of the sample was plated onto tryptic soy agar (TSA) and cefsulodin–irgasan–novobiocin (CIN) agar plates and incubated at 28 °C for 48 h. Figure S2. Mean depth across the Yersinia pestis CO92 chromosome after whole-genome amplification (WGA) and target enrichment (TE). Mean depth was calculated in 10 kb windows using specifically mapped to the Y. pestis Co92 chromosome for samples #24-2, #24-5, #24-8, and #24-10. Mb, megabase. Table S1. Threshold cycle (Ct) values from real-time PCR for Yersinia pestis target genes using 1/10 serially diluted DNA standards. Table S2. Read proportions determined with taxonomic profiling using the All Living Organisms (ALO) database and mitochondrial DNA for the four samples. Table S3. Proportion of Yersinia pestis CO92 reference-matched reads obtained under whole-genome amplification (WGA) versus target enrichment (TE) conditions. Table S4. Proportion of 25 MLVA loci of Yersinia pestis CO92 matched reads obtained under whole-genome amplification (WGA) versus target enrichment (TE) conditions. Table S5. MLVA profile results for 25 loci under whole-genome amplification (WGA) and target enrichment (TE).

## Abbreviations

ALO	All Living Organisms
BLAST	Basic Local Alignment Search Tool
CIN	Cefsulodin–irgasan–novobiocin
C_t_	Threshold cycle
CY5	Cyanine 5
FAM	6-carboxyfluorescein
i-EBQ	Incorporated an internal Bioneer quencher
LOD	Limit of detection
MLST	Multilocus sequence typing
MLVA	Multilocus variable number tandem repeat analysis
mtDNA	Mitochondrial DNA
NCBI	National Center for Biotechnology Information
NGS	Next-generation sequencing
PCR	Polymerase chain reaction
SNPs	Single-nucleotide polymorphisms
TEX	Texas Red
TSA	Tryptic soy agar
VNTR	Variation in the number of tandem repeats
WGA	Whole-genome amplification
wgMLST	Whole-genome multilocus sequence typing
WGS	Whole-genome sequencing
