# Supplementary material for: Typing of Yersinia pestis in Challenging Forensic Samples Through Targeted Next-Generation Sequencing of Multilocus Variable Number Tandem Repeat Regions
Source: Microorganisms. 2025 Oct 7;13(10):2320. doi: 10.3390/microorganisms13102320 (PMC12566482; doi:10.3390/microorganisms13102320)
Supplement: Supplementary file 1 [file microorganisms-13-02320-s001.zip › Supplementary_Information_Table S1.pdf]

Table S1. Threshold cycle (C_t) values from real-time PCR for *Yersinia pestis* target genes using 1/10 serially diluted DNA standards

**Copy number**	***yihN***	***caf1***	***pla***
10^5	22.42	21.17	24.47
10^4	25.82	24.50	27.61
10^3	29.65	27.19	31.15
10^2	33.03	31.11	34.91
10^1	36.61	34.53	38.89
10^0	N.D.	36.67*	N.D.

Note: C_t values represent the mean of values from triplicate real-time PCR reactions. *C_t value detected in two of three replicate reactions. PCR, polymerase chain reaction; N.D., not detected.
