# Supplementary material for: Typing of Yersinia pestis in Challenging Forensic Samples Through Targeted Next-Generation Sequencing of Multilocus Variable Number Tandem Repeat Regions
Source: Microorganisms. 2025 Oct 7;13(10):2320. doi: 10.3390/microorganisms13102320 (PMC12566482; doi:10.3390/microorganisms13102320)
Supplement: Supplementary file 1 [file microorganisms-13-02320-s001.zip › Supplementary_Information_Table S2.pdf]

Table S2. Read proportions determined with taxonomic profiling using the All Living Organisms (ALO) database and mitochondrial DNA for the four samples

**Sample**	**Unmapped**	**ALO**	**mtDNA**
#24-2	9.5%	90.0%	0.5%
#24-5	15.1%	84.2%	0.7%
#24-8	21.7%	9.0%	69.3%
#24-10	15.1%	84.6%	0.3%

mtDNA, mitochondrial DNA
