# Supplementary material for: Typing of Yersinia pestis in Challenging Forensic Samples Through Targeted Next-Generation Sequencing of Multilocus Variable Number Tandem Repeat Regions
Source: Microorganisms. 2025 Oct 7;13(10):2320. doi: 10.3390/microorganisms13102320 (PMC12566482; doi:10.3390/microorganisms13102320)
Supplement: Supplementary file 1 [file microorganisms-13-02320-s001.zip › Supplementary_Information_Table S3.pdf]

Table S3. Proportion of *Yersinia pestis* CO92 reference-matched reads obtained under whole genome amplification (WGA) versus target enrichment (TE) conditions

Sample	Enrichment	Total reads	Reference matched reads	Percentage
#24-2	WGA	8,132,494	39,163	0.48%
	TE	396,716	281,334	70.9%
#24-5	WGA	10,379,556	8,817,583	85.0%
	TE	501,550	393,294	78.4%
#24-8	WGA	11,094,794	384,618	3.47%
	TE	566,767	445,959	78.7%
#24-10	WGA	7,643,894	367	0.005%
	TE	165,033	154,001	93.3%
