# Supplementary material for: Typing of Yersinia pestis in Challenging Forensic Samples Through Targeted Next-Generation Sequencing of Multilocus Variable Number Tandem Repeat Regions
Source: Microorganisms. 2025 Oct 7;13(10):2320. doi: 10.3390/microorganisms13102320 (PMC12566482; doi:10.3390/microorganisms13102320)
Supplement: Supplementary file 1 [file microorganisms-13-02320-s001.zip › Supplementary_Information_Table S4.pdf]

Table S4. Proportion of 25 MLVA loci of *Yersinia pestis* CO92 matched reads obtained under whole genome amplification (WGA) versus target enrichment (TE) conditions

Sample	Enrichment	Total reads	25 MLVA loci matched reads	Percentage
#24-2	WGA	8,132,494	33	0.0004%
	TE	396,716	40,291	10.15%
#24-5	WGA	10,379,556	6,411	0.062%
	TE	501,550	43,757	8.72%
#24-8	WGA	11,094,794	258	0.002%
	TE	566,767	44,166	7.79%
#24-10	WGA	7,643,894	0	0%
	TE	165,033	25,395	15.39%
