# Supplementary material for: Typing of Yersinia pestis in Challenging Forensic Samples Through Targeted Next-Generation Sequencing of Multilocus Variable Number Tandem Repeat Regions
Source: Microorganisms. 2025 Oct 7;13(10):2320. doi: 10.3390/microorganisms13102320 (PMC12566482; doi:10.3390/microorganisms13102320)
Supplement: Supplementary file 1 [file microorganisms-13-02320-s001.zip › Supplementary_Information_Table S5.pdf]

Table S5. MLVA profile results for 25 loci under whole genome amplification (WGA) and target enrichment (TE)

Name	#24-2		#24-5		#24-8		#24-10		Validated profiles
	WGA	TE	WGA	TE	WGA	TE	WGA	TE	
YPO0120ms01	N.D.	6	6	6	6	6	N.D.	6	6
YPO1290ms04	N.D.	6	6	6	5	6	N.D.	6	6
YPO1935ms05	3	10	10	10	9	10	N.D.	10	10
YPO2769ms06	1	3	3	3	3	3	N.D.	3	3
YPO2916ms07	N.D.	7	7	7	8	7	N.D.	7	7
YPO3057ms09	17	23	23	23	23	23	N.D.	23	23
YPO0559ms15	N.D.	10	10	10	12	10	N.D.	10	10
YPO1814ms20	N.D.	8	8	8	7	8	N.D.	8	8
YPO1895ms21	2	9	9	9	8	9	N.D.	9	9
YPO4042ms35	9	9	9	9	10	9	N.D.	9	9
YPO4425ms38	3	6	6	6	6	6	N.D.	6	6
YPO0581ms40	3	8	8	8	8	8	N.D.	8	8
YPO0718ms41	N.D.	5	5	5	5	5	N.D.	5	5
YPO1018ms44	N.D.	7	7	7	7	7	N.D.	7	7
YPO1108ms45	2	6	6	6	6	6	N.D.	6	6
YPO1335ms46	9	24	24	24	22	24	N.D.	24	24
YPO2058ms51	N.D.	2	2	2	2	2	N.D.	2	2
YPO2612ms54	5	7	7	7	7	7	N.D.	7	7

YPO3060ms56	6	9	9	9	9	9	N.D.	9	9
YPO4280ms62	N.D.	11	11	11	17	11	N.D.	11	11
YPO1118ms69	3	5	5	5	3	5	N.D.	5	5
YPO1580ms70	N.D.	6	6	6	6	6	N.D.	6	6
YPO1925ms71	N.D.	5	5	5	3	5	N.D.	5	5
YPO3236ms73	2	5	5	5	4	5	N.D.	5	5
YPO3245ms74	N.D.	8	8	8	8	8	N.D.	8	8

MLVA, multilocus variable number tandem repeat analysis; N.D., Not Determined.
