# Supplementary figures and images for: Typing of Yersinia pestis in Challenging Forensic Samples Through Targeted Next-Generation Sequencing of Multilocus Variable Number Tandem Repeat Regions
Source: Microorganisms. 2025 Oct 7;13(10):2320. doi: 10.3390/microorganisms13102320 (PMC12566482; doi:10.3390/microorganisms13102320)

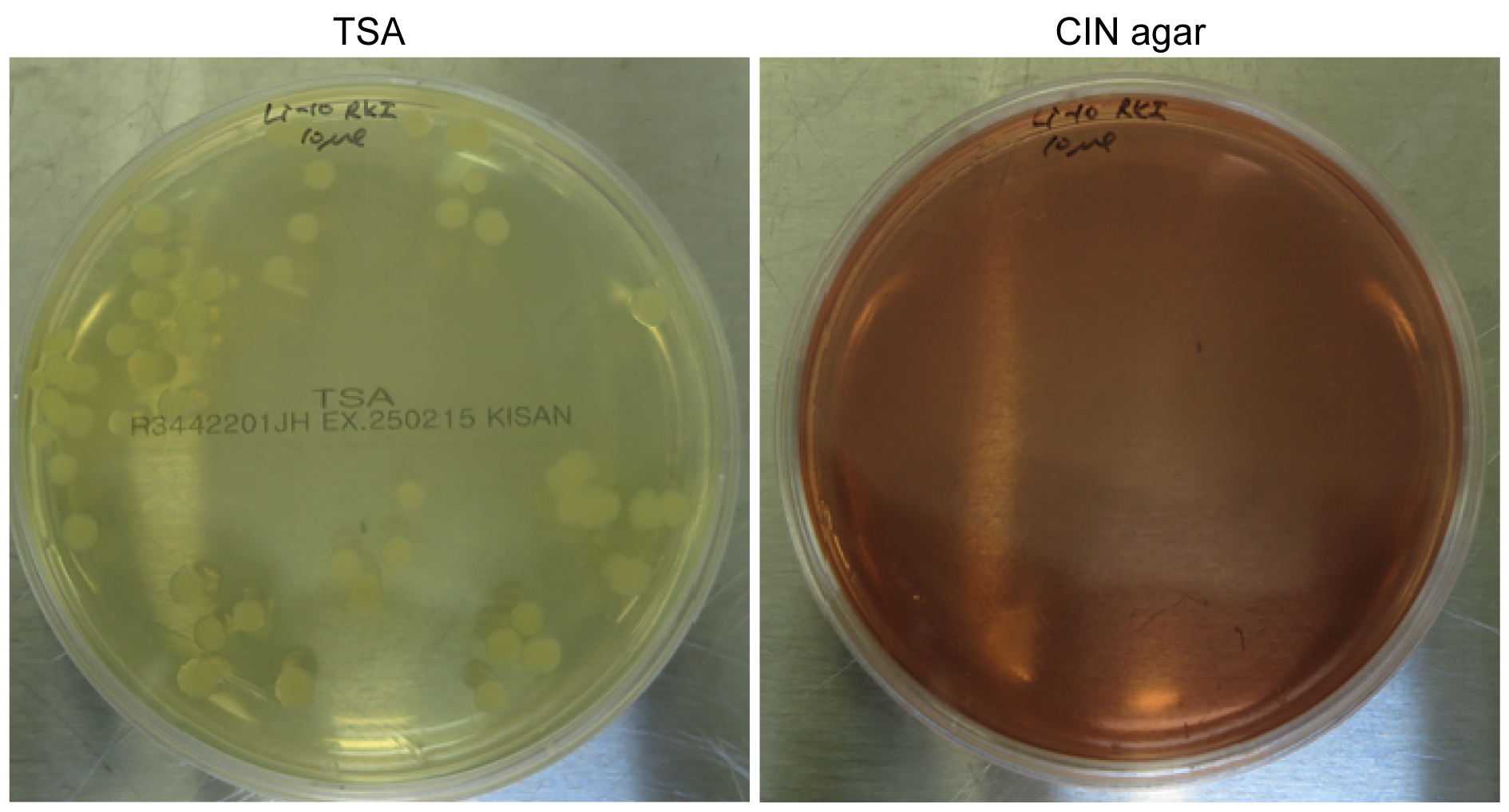

Supplement: Supplementary file 1 [file microorganisms-13-02320-s001.zip › Supplementary_Information_Figure S1.tif]

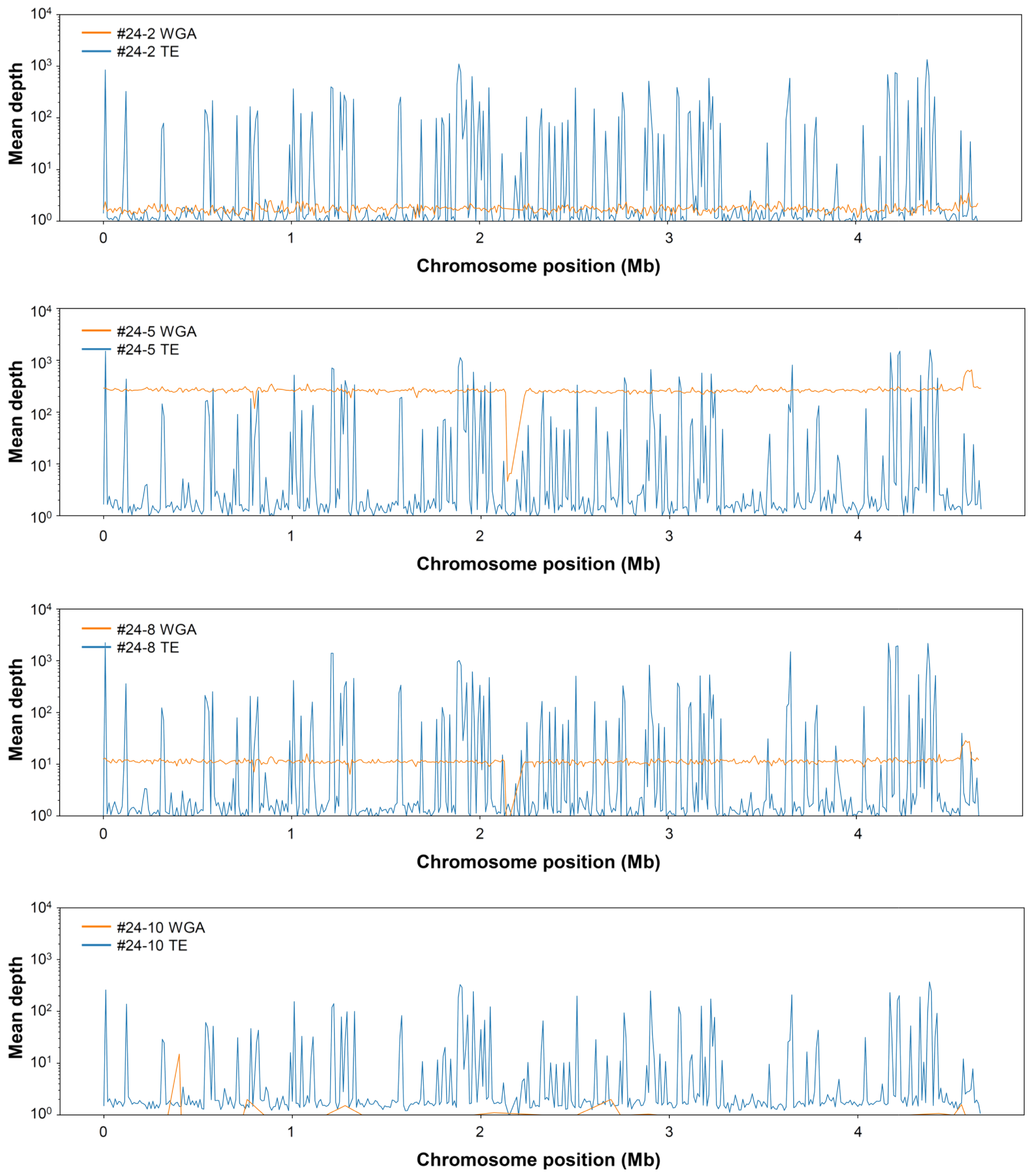

Supplement: Supplementary file 1 [file microorganisms-13-02320-s001.zip › Supplementary_Information_Figure S2.tif]
